# SPECT/CT imaging of chemotherapy-induced tumor apoptosis using ^99m^Tc-labeled dendrimer-entrapped gold nanoparticles

**DOI:** 10.1080/10717544.2018.1474968

**Published:** 2018-06-05

**Authors:** Yan Xing, Jingyi Zhu, Lingzhou Zhao, Zhijuan Xiong, Yujie Li, San Wu, Gitasha Chand, Xiangyang Shi, Jinhua Zhao

**Affiliations:** a Department of Nuclear Medicine, Shanghai General Hospital of Nanjing Medical University, Shanghai, People’s Republic of China;; b Department of Nuclear Medicine, Shanghai General Hospital, Shanghai Jiao Tong University School of Medicine, Shanghai, People’s Republic of China;; c State Key Laboratory for Modification of Chemical Fibers and Polymer Materials, College of Chemistry, Chemical Engineering and Biotechnology, Donghua University, Shanghai, People’s Republic of China;; d School of Pharmaceutical Science, Nanjing Tech University, Nanjing, People’s Republic of China

**Keywords:** Dendrimers, duramycin, apoptosis, chemotherapy, SPECT/CT imaging

## Abstract

Non-invasive imaging of apoptosis in tumors induced by chemotherapy is of great value in the evaluation of therapeutic efficiency. In this study, we report the synthesis, characterization, and utilization of radionuclide technetium-99m (^99m^Tc)-labeled dendrimer-entrapped gold nanoparticles (Au DENPs) for targeted SPECT/CT imaging of chemotherapy-induced tumor apoptosis. Generation five poly(amidoamine) (PAMAM) dendrimers (G5.NH_2_) were sequentially conjugated with 1,4,7,10-tetraazacyclododecane-1,4,7,10-tetraacetic acid (DOTA), polyethylene glycol (PEG) modified duramycin, PEG monomethyl ether, and fluorescein isothiocyanate (FI) to form the multifunctional dendrimers, which were then utilized as templates to entrap gold nanoparticles. Followed by acetylation of the remaining dendrimer surface amines and radiolabeling of ^99m^Tc, the SPECT/CT dual mode nanoprobe of tumor apoptosis was constructed. The developed multifunctional Au DENPs before and after ^99m^Tc radiolabeling were well characterized. The results demonstrate that the multifunctional Au DENPs display favorable colloidal stability under different conditions, own good cytocompatibility in the given concentration range, and can be effectively labeled by ^99m^Tc with high radiochemical stability. Furthermore, the multifunctional nanoprobe enables the targeted SPECT/CT imaging of apoptotic cancer cells *in vitro* and tumor apoptosis after doxorubicin (DOX) treatment in the established subcutaneous tumor model *in vivo*. The designed duramycin-functionalized Au DENPs might have the potential to be employed as a nanoplatform for the detection of apoptosis and early tumor response to chemotherapy.

## Introduction

Early and accurate monitoring of tumor response to anticancer treatments can greatly assist clinicians to identify non-responsive patients and pair them with more effective and individualized therapies. Currently, treatment responses are typically measured by estimating tumor size using computed tomography (CT) or magnetic resonance (MR) imaging (Eisenhauer et al., 2009; Wang et al., 2017). However, a significant decrease in tumor size only occurs several weeks to months after initial treatment. This makes current conventional imaging techniques limited and insufficient in the early evaluation of its therapeutic effects (Goffin et al., 2005). Molecular imaging techniques such as positron emission tomography (PET) and single photon emission computed tomography (SPECT), based on noninvasive imaging of tumor physiology and metabolism that precede apparent morphologic changes, can provide sensitive and specific approaches for monitoring early treatment in oncology (Brindle, 2008; Garin et al., 2012; Vaidya et al., 2012; Lin et al., 2017; Zhao et al., 2017). Furthermore, these molecular imaging modalities can be integrated with CT or MR to compensate for the lack of anatomical information, leading to more precise evaluation (Delbeke et al., 2009; Kao et al., 2012; Palmedo et al., 2014; Koh et al., 2016). Taking SPECT/CT as an example, this technique can simultaneously acquire functional-metabolic information from SPECT and detailed anatomical information from CT, which has been confirmed as a powerful tool in disease diagnosis, prognosis and therapy monitoring. However, development of desired contrast agents for efficient SPECT/CT imaging remains a challenge.

Nanotechnology offers great advantages to construct various contrast agents for different imaging modalities, in particular radionuclide-labeled nanoparticles (NPs) for molecular imaging (Xing et al., 2014; Li et al., 2016; Wang et al., 2017; Zhou et al., 2018). It is worth noting that dendrimers can effectively load different kinds of radionuclides and other imaging moieties for hybrid imaging such as PET/CT and SPECT/CT, due to its unique features including precise molecular structures, multiple surface functional groups, convenient surface modifications, and excellent biocompatibility (Zhao et al., 2017). In the construction of dendrimer-based nanoprobes for SPECT/CT imaging, it is required to label SPECT radionuclides within the NP system. Commonly, technetium-99m (^99m^Tc) is an ideal candidate due to its low-energy γ-ray (140 keV), proper half-life (6.02 h), inexpensiveness, and commercial availability (Mariani et al., 2010). Besides, the ^99m^Tc radiolabeling of dendrimers can be readily achieved with high yield through bifunctional chelators (e.g. DOTA) modified onto dendrimers. For CT imaging, dendrimers are also well known as appropriate carriers to load Au NPs that possess a higher X-ray attenuation property compared with the clinical iodine-based small molecules (e.g. Omnipaque). For instance, dendrimers have been employed as templates or stabilizers to synthesize Au NPs for blood pool, major organ, and tumor imaging (Qiao & Shi, 2015; Wei et al., 2016; Xiong et al., 2017). These dendrimer-based Au NPs possess remarkable properties, including improved diagnostic effects, extended blood circulation time, and controllable behaviors *in vivo*. Therefore, through simultaneous interior entrapment of Au NPs and peripheral radiolabeling of ^99m^Tc, dendrimer-based SPECT/CT imaging nanoprobes can been developed. Moreover, these dual-mode nanoprobes can be further functionalized with targeting molecules to obtain desired specificity *in vivo*. Currently, many targeting molecules have been successfully modified with dendrimer-based NPs for targeted diagnosis and therapy in a wide variety of tumors (Zhao et al., 2015; Cheng et al., 2016; Li et al., 2016; Wang et al., 2016). However, there are few examples to utilize dendrimers to build contrast agents for the evaluation of early tumor response to therapy, especially integration of SPECT and CT imaging components within a single nanoparticle system for dual modality imaging.

In our recent work, we have demonstrated that PAMAM dendrimers can be used to entrap Au NPs and efficiently labeled with ^99m^Tc for tumor dual mode SPECT/CT imaging applications (Li et al., 2016; Wen et al., 2017; Xu et al., 2017). *Via* different surface modification, the formed nanoprobes could be afforded with different behaviors and biodistributions *in vivo*, enabling the preferential SPECT/CT imaging of various organs, lymph nodes, or tumor tissues. The previous successes of dendrimer-based multifunctional NPs inspire us to speculate that PAMAM dendrimers are also able to be explored as a nanoplatform to develop SPECT/CT dual mode nanoprobes for early detection of apoptosis in tumors. Duramycin, an important peptide with high affinity and selectivity for phosphatidylethanolamine (PE) that externalizes to cell surface during apoptosis, has been developed as a targeting ligand for SPECT imaging of cell apoptosis *in vivo* (Elvas et al., 2016; Luo et al., 2016; Elvas et al., 2017).

In this work, we designed and synthesized the ^99m^Tc-labeled multifunctional dendrimer-entrapped Au NPs functionalized with duramycin to detect tumor apoptosis after chemotherapy using SPECT/CT imaging. Firstly, amine-terminated G5 PAMAM dendrimers modified with DOTA mono-N-hydroxysuccinimide ester (DOTA-NHS), polyethylene glycol (PEG) linked duramycin, PEG monomethyl ether with one end of carboxyl group (*m*PEG-COOH), and fluorescein isothiocyanate (FI) to form the multifunctional dendrimer templates, and then the templates were utilized to entrap Au NPs. Followed by acetylation of the remaining dendrimer terminal amines and radiolabeling with ^99m^Tc *via* DOTA chelation, {(Au^0^)_200_-G5.NHAc-^99m^Tc-DOTA-FI-*m*PEG-(PEG-duramycin)} DENPs (^99m^Tc-duramycin-Au DENPs) were formed. The formed multifunctional Au DENPs before and after ^99m^Tc labeling were well-characterized *via* different techniques, including the structure, X-ray attenuation coefficient, colloidal stability under different pH and temperature conditions, cytocompatibility at an Au concentration up to 200 μM, and radiochemical stability *in vitro*. Furthermore, the developed ^99m^Tc-duramycin-Au DENPs were utilized for targeted SPECT/CT imaging of apoptotic cancer cells *in vitro* and tumor apoptosis *in vivo* in a xenografted tumor model after doxorubicin (DOX) treatment. To the best of our knowledge, this study is the first to report the development of dendrimer-based dual mode nanoprobe for SPECT/CT imaging of tumor cell apoptosis.

## Materials and methods

### Materials

G5.NH_2_ PAMAM dendrimers were obtained from Dendritech (Midland, MI). Duramycin was purchased from Beijing Abace Biology Co., Ltd. (Beijing, China). DOTA, 1-ethyl-3-(3-(dimethylamino)propyl) carbodiimide hydrochloride (EDC), stannous chlorides (SnCl_2_), sodium borohydride (NaBH_4_), and cell counting kit-8 (CCK-8) were supplied by Sigma-Aldrich (St. Louis, MO). PEG monomethyl ether with one end of carboxyl group (*m*PEG–COOH, *M*_w_ = 5000), PEG with one end of amine group and the other end of carboxyl group (NH_2_–PEG–COOH, *M*_w_ = 5000), cellulose dialysis membranes, and phosphate buffered saline (PBS) were obtained from Shanghai Yanyi Biotechnology Corporation (Shanghai, China). ^99m^Tc-pertechnetate (Na^99m^TcO_4_) was purchased from Shanghai GMS Pharmaceutical Co., Ltd. (Shanghai, China). Disposable PD-10 desalting columns, DOX, C6 glioma cells, fetal bovine serum (FBS), RPMI 1640 medium, penicillin, and streptomycin were procured from Shanghai dobio CO., Ltd. (Shanghai, China). Acetic anhydride (Ac_2_O), HAuCl_4_·4H_2_O, triethylamine (TEA), and all other chemicals and solvents were supplied by Sinopharm Chemical Reagent Co., Ltd (Shanghai, China).

### Synthesis of {(Au^0^)_200_-G5.NHAc-^99m^Tc-DOTA-FI-*m*PEG-(PEG-duramycin)} DENPs

Duramycin-conjugated dendrimers were synthesized according to our previously reported method (Zhao et al., 2015; Luo et al., 2016). Firstly, duramycin was modified with NH_2_–PEG–COOH to form duramycin–PEG–COOH. Briefly, duramycin (10 mg) was activated with EDC (0.95 mg) in DMSO (5 mL) at room temperature for 3 h. Then the EDC-activated duramycin was dropwise added into the DMSO solution (5 mL) of NH_2_–PEG–COOH (16.6 mg) under vigorous magnetic stirring at room temperature for 24 h. Finally, the reaction mixture was dialyzed against phosphate buffered saline (PBS, three times, 2 L) and water (three times, 2 L) through a dialysis membrane with an MWCO of 3500 for 3 days to remove the excess reactants, and the product duramycin–PEG–COOH was obtained.

Secondly, DOTA–NHS, duramycin–PEG–COOH, *m*PEG–COOH and FI were sequentially conjugated with G5 dendrimers to prepare G5.NH_2_-DOTA-FI-*m*PEG-(PEG-duramycin). In brief, DOTA–NHS ester (2.9 mg) dissolved in 2 mL DMSO was dropwise added to the 5 mL DMSO solution of G5.NH_2_ (10 mg) under vigorous magnetic stirring. The reaction mixture was incubated at room temperature for 24 h to yield raw product of G5.NH_2_-DOTA. Then, the duramycin–PEG–COOH (25.9 mg, 5 mL DMSO) activated by EDC (17.7 mg, 2 mL DMSO) was mixed with the DMSO solution of G5.NH_2_-DOTA under continual stirring for 3 days to get G5.NH_2_-DOTA-(PEG-duramycin). The *m*PEG–COOH (23.1 mg, 2 mL DMSO) pre-activated by EDC (17.7 mg, 2 mL DMSO) was added into the G5.NH_2_-DOTA-duramycin mixture solution under stirring vigorously for 3 days, followed by mixing with FI (1.05 mg, 2 mL) to react for 24 h, and then the G5.NH_2_-DOTA-FI-*m*PEG-(PEG-duramycin) was synthesized. At last, the G5.NH_2_-DOTA-FI-*m*PEG-(PEG-duramycin) mixture was extensively dialyzed against PBS (three times, 2 L) and water (three times, 2 L) for another 3 days through a dialysis membrane with an MWCO of 14 000. The dialysis liquid was lyophilized to acquire the product of G5.NH_2_-DOTA-FI-*m*PEG-(PEG-duramycin).

Lastly, duramycin-conjugated Au DENPs were synthesized using the prepared G5.NH_2_-DOTA-FI-*m*PEG-(PEG-duramycin) as templates. Typically, 1.7 mL of HAuCl_4_ solution (30 mg/mL) with the dendrimers/Au salt molar ratio at 1:200 was mixed with the synthesized G5.NH_2_-DOTA-FI-*m*PEG-(PEG-duramycin) (86.0 mg, 10 mL water) under vigorous stirring for 0.5 h. Followed by the addition of cold NaBH_4_ water solution (10.0 mg/mL, 1.4 mL) under stirring for 2 h, the raw product of {(Au^0^)_200_-G5.NH_2_-DOTA-FI-*m*PEG-(PEG-duramycin)} DENPs was formed. The raw product was then acetylated by the sequential addition of TEA (57.4 μL) under vigorous magnetic stirring for 0.5 h, and the Ac_2_O (32.5 μL) under stirring for 24 h to obtain the final product of {(Au^0^)_200_-G5.NHAc-DOTA-FI-*m*PEG-(PEG-duramycin)} DENPs (duramycin-Au DENPs). The excess of reactants and byproducts were removed by dialysis against PBS (three times, 2 L) and water (five times, 2 L) for 3 days through a dialysis membrane with an MWCO of 14,000. After a lyophilization process, the duramycin-conjugated multifunctional Au DENPs were acquired. For comparison, the Au DENPs without duramycin modification were also synthesized using the same method under the same experimental conditions. The intermediate products of G5.NH_2_-DOTA, G5.NH_2_-DOTA-duramycin and G5.NH_2_-DOTA-FI-*m*PEG-(PEG-duramycin) were gathered and analyzed by calculating the number of DOTA, duramycin, *m*PEG, and FI ligands modified onto each dendrimer.

### ^99m^Tc radiolabeling of {(Au^0^)_200_-G5.NHAc-DOTA-FI-*m*PEG-(PEG-duramycin)} DENPs

The duramycin-Au DENPs were labeled with ^99m^Tc through DOTA ligands according to the protocol described in our previous work (Luo et al., 2016). Briefly, ^99m^Tc-pertechnetate solution (740 MBq, 200 μL) was added to a 5-mL vial containing SnCl_2_ (50 μg) and duramycin-Au DENPs (200 μg) dissolved in 250 μL of PBS (0.1 M, pH = 7.2–7.4) with continuous stirring. After incubation for 30 min at room temperature, the reaction was stopped and the reaction mixture was eluted by PD-10 desalting columns using PBS solution as the mobile phase. The eluted fractions containing ^99m^Tc-duramycin-Au DENPs were collected and its radioactivity was recorded using a CRC-15R radioisotope dose calibrator (Capintec, Inc., Ramsey, NJ). The ^99m^Tc-Au DENPs without duramycin modification was also prepared under the same experimental conditions for comparison.

### Flow cytometry analysis

Flow cytometry assay was performed to investigate the targeting specificity of duramycin-Au DENPs toward apoptotic tumor cells. Briefly, 2 × 10^5^ C6 cells were seeded into each well of a 6-well plate and were treated with 2 μM DOX for 24 h to induce cell apoptosis, and normal C6 cells served as a control. After overnight incubation, the medium was replaced with 1 mL of fresh DMEM containing the duramycin-Au DENPs or Au DENPs at the Au concentrations of 0.4 μM and 4 μM, respectively. After 2 h, the cells were washed three times with PBS, trypsinized, resuspended in PBS containing 0.1% bovine serum albumin, and measured using a Becton Dickinson FACScan analyzer. Dickinson FACScan analyzer (Franklin Lakes, NJ). Approximately 10,000 cells were recorded in the FL1-fluorescence channel, and the mean fluorescence of the gated viable cells was analyzed.

### Confocal microscopy

The cellular uptake of duramycin-Au DENPs by apoptotic C6 cells was observed with confocal microscopy (Carl Zeiss LSM 700, Jena, Germany). Briefly, coverslips were pretreated according to our previous work (Zhao et al., 2015). 5 × 10^4^ C6 cells were seeded into each well of a 12-well plate and cultured overnight to allow the cells to attach onto the coverslips. Then these cells were treated with 2 μM DOX for 24 h to induce apoptosis. The medium was replaced with 1 mL of fresh medium containing PBS (control), Au DENPs and duramycin-Au DENPs ([Au] = 4 μM), respectively. The cells were incubated at 37 °C and 5% CO_2_ for 2 h, rinsed with PBS, fixed with glutaraldehyde (2.5%) at 4 °C for 15 min, and then counterstained with Hoechst 33342 (1 μg/mL) at 37 °C for 20 min according to standard procedures. The FI fluorescence was excited with a 488 nm argon blue laser and a 505–525 nm barrier filter was applied to collect the FI emission. Samples were scanned using a 63× oil-immersion objective lens, and the optical section thickness was set at 5 mm.

### SPECT and CT imaging of apoptotic C6 cells *in vitro*

For SPECT imaging *in vitro*, the apoptotic and normal C6 cells cultured under the same conditions described above were incubated with the ^99m^Tc-duramycin-Au DENPs or ^99m^Tc-Au DENPs at different radiodoses (25, 50, 100, 200, and 400 μCi/mL) for 4 h. Then, the cells were treated under the above conditions and scanned according to our previous work (Li et al., 2016). Similarly, apoptotic and normal C6 cells were incubated with fresh medium containing PBS, duramycin-Au DENPs, or Au DENPs at different Au concentrations (20, 40, 60, 80, and 100 μM) for 3 h. After appropriate treatment according to our previous work (Zhou et al., 2018), the cells were imaged by a GE Discovery STE PET/CT system with a tube voltage of 100 kV, an electrical current of 220 mA, and a slice thickness of 1.25 mm.

### SPECT and CT imaging *in vivo*

Animal experiments were approved by the ethical committee of Shanghai General Hospital. Before *in vivo* imaging experiments, the mice were anesthetized with pentobarbital sodium (40 mg/kg) and randomly divided into experimental and control groups (five mice per group). For SPECT imaging, we intravenously injected a PBS solution of ^99m^Tc-duramycin-Au DENPs ([^99m^Tc] = 740 MBq/mL, 100 μL) to the mice in the experimental group and ^99m^Tc-Au DENPs at the same dose to the control group. SPECT images were acquired at 0.5, 2, 4, 6, 8, and 12 h post-injection using an Infinia SPECT scanner equipped with a Xeleris Workstation and Low Energy General Purpose collimator (GE Inc., Fairfield, CT). At 8 h post-injection, one mouse from each respective group was sacrificed. The major organs (heart, liver, spleen, lung, and kidneys) and tumors were removed immediately and their relative radioactivity ratios were recorded by analyzing the regions of interest. For comparison, the SPECT images were first obtained before the mice were treated with 40 mg/kg DOX. After 3 days of DOX treatment, the SPECT studies were performed again in the same procedure. For CT imaging, the DOX treated tumor-bearing nude mice were intravenously injected with the duramycin-Au DENPs or Au DENPs ([Au] = 0.08 M, in 0.10 mL saline) and scanned before and at different time points post-injection (0.5, 2, 4, 6, 8, and 12 h) by the same CT system.

### H&E staining and TUNEL assay

After the imaging experiments, one mouse from each group was sacrificed and the tumors and major organs were extracted. According to the standard procedure of hematoxylin and eosin (H&E) staining, the tumors and organs were fixed, embedded, sectioned, stained, and observed. The tumor tissue apoptosis of mice after DOX treatment was further confirmed using a terminal deoxynucleotidyl transferase dUTP nick end labeling (TUNEL) method by apoptotic detection kit (Roche, Basel, Switzerland) according to the literature (Zhao et al., 2015). Through the treatments of fixation, dehydration, paraffin-embedment, sectioning, and staining using a TUNEL kit, the tumor sections were finally observed.

### Statistical analysis

The significance of the experimental data was assessed by one-way ANOVA statistical analysis method. A value of .05 was considered to be significant, and the data were marked with (∗∗) for *p* < .01 and (∗∗∗) for *p* < .001, respectively.

## Results and discussion

### Synthesis and characterization of the {(Au^0^)_200_-G5.NHAc-^99m^Tc-DOTA-FI-*m*PEG-(PEG-duramycin)} DENPs

**Figure 1.**
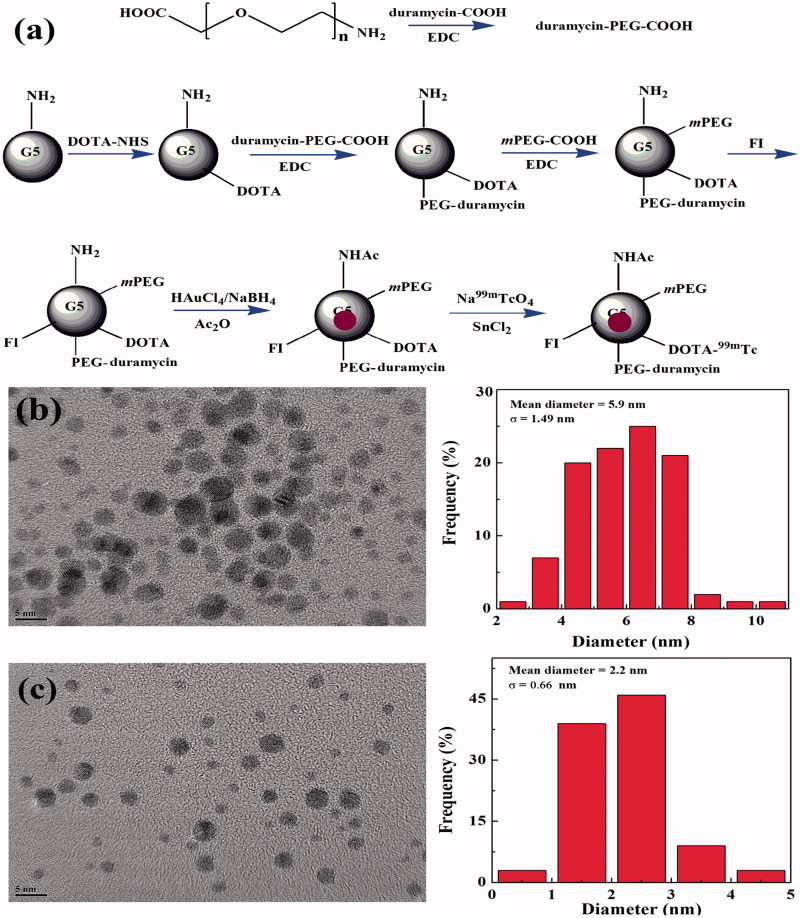
Schematic illustration of the synthesis of the ^99m^Tc-duramycin-Au DENPs (a). TEM image and size distribution of the duramycin-Au DENPs (b) and Au DENPs (c).

^99m^Tc-labeled multifunctional Au DENPs were designed and synthesized for targeted SPECT/CT imaging of tumor apoptosis after chemotherapy. In this study, amine-terminated G5 PAMAM dendrimers were sequentially modified with DOTA, PEGylated duramycin, *m*PEG-COOH, and FI to form multifunctional dendrimer templates. Then the templates were used to entrap Au NPs, followed by acetylation of the remaining dendrimer terminal amines and radiolabeling of ^99m^Tc, and then the ^99m^Tc-duramycin-Au DENPs were manufactured (Figure 1(a)).

First, the DOTA-modified G5.NH_2_ was synthesized and characterized *via*^1^H NMR spectroscopy. The number of DOTA conjugated onto each G5.NH_2_ dendrimer was calculated using the subtractive assay as our previous studies due to the –CH_2_– proton signals of DOTA overlap with those of the ethylene backbone on dendrimers (Chen et al., 2013; Wen et al., 2013). The number of DOTA was calculated to be 8.9 (Figure S1(a,b)). The duramycin-PEG-COOH was also characterized *via*^1^H NMR spectroscopy. According to the characteristic peaks of phenyl on duramycin located at 7.3–7.8 ppm and characteristic peaks of NH_2_–PEG–COOH located at 3.5–3.8 ppm, the average number of duramycin attached onto each NH_2_–PEG–COOH was calculated to be 0.3 *via*^1^H NMR integration (Figure S1(c)). The similar method was utilized to calculate the number of duramycin conjugated onto each G5.NH_2_ by integration the characteristic peaks of duramycin located at 7.3–7.8 ppm and characteristic peaks of G5.NH_2_ located at 2.0–3.0 ppm. The average numbers of duramycin attached onto each G5 dendrimer was estimated to be 3.8 (Figure S1(d)). The *m*PEG–COOH was then conjugated onto G5 dendrimers, it can be calculated that the average number of PEG (NH_2_–PEG–COOH and *m*PEG–COOH) modified onto each G5.NH_2_ is 19.2 (Figure S1(e)). And the number of FI conjugated onto each G5 dendrimer was estimated to be 6.2 by integration the characteristic peaks of FI moieties (Figure S1(e)). The G5-NH_2_-DOTA-FI-*m*PEG without duramycin conjugation was also characterized *via*^1^H NMR spectroscopy. There are 19.5 *m*PEG-COOH modified onto each G5.NH_2_, which is similar to PEG modification on the G5.NH_2_-DOTA-FI-*m*PEG-(PEG-duramycin) dendrimer (Figure S1(f)).

Second, the prepared G5.NH_2_-DOTA-FI-*m*PEG-(PEG-duramycin) dendrimers were used as templates to synthesize duramycin-Au DENPs. The Au salt/dendrimer molar ratio was selected to be 200:1, which was similar to our previous work (Wen et al., 2017). The inductively coupled plasma-optical emission spectroscopy (ICP-OES) was utilized to determine the Au content within the dendrimers. The results suggest that the added Au(III) salt has been completely reduced to Au(0) in the duramycin-Au DENPs and Au DENPs, and their average numbers of Au atoms per G5 dendrimer are extremely close to the initial Au salt/dendrimer molar ratio. The synthesized duramycin-Au DENPs displayed a strong characteristic surface plasmon resonance peak at 540 nm in the UV–vis spectrum, which demonstrates the formation of Au NPs (Figure S2(a)). The remaining dendrimer terminal amine groups of Au DENPs were then acetylated by Ac_2_O. The formed duramycin-Au DENPs and Au DENPs have a surface potential of 11.93 and −0.11 mV, respectively, indicating the successful surface acetylation reactions.

TEM images reveal that the Au core NPs possess a close-to-spherical shape with quite a small size and uniform size distribution (Figure 1(b,c)), and the mean Au core diameters of the duramycin-Au DENPs and Au DENPs are 5.9 and 2.2 nm, respectively. The hydrodynamic sizes of both Au DENPs dispersed in water were also analyzed by DLS, and the data show that the duramycin-Au DENPs and Au DENPs have a hydrodynamic size of 327.0 and 287.9 nm, respectively with a quite low polydispersity index (Table S1).

Last, ^99m^Tc was labeled onto the surface of the duramycin-Au DENPs and Au DENPs *via* DOTA chelation. Instant thin-layer chromatography (ITLC) data revealed that the efficiency of labeling ^99m^Tc onto the duramycin-Au DENPs or Au DENPs was 60.4 ± 5.4 and 64.5 ± 6.8% (*n* = 3), respectively. Both the radiochemical purities of ^99m^Tc-duramycin-Au DENPs and ^99m^Tc-Au DENPs after purification were above 99%.

### Specific targeting of {(Au^0^)_200_-G5.NHAc-DOTA-FI-*m*PEG-(PEG-duramycin)} DENPs to apoptotic cancer cells

**Figure 2.**
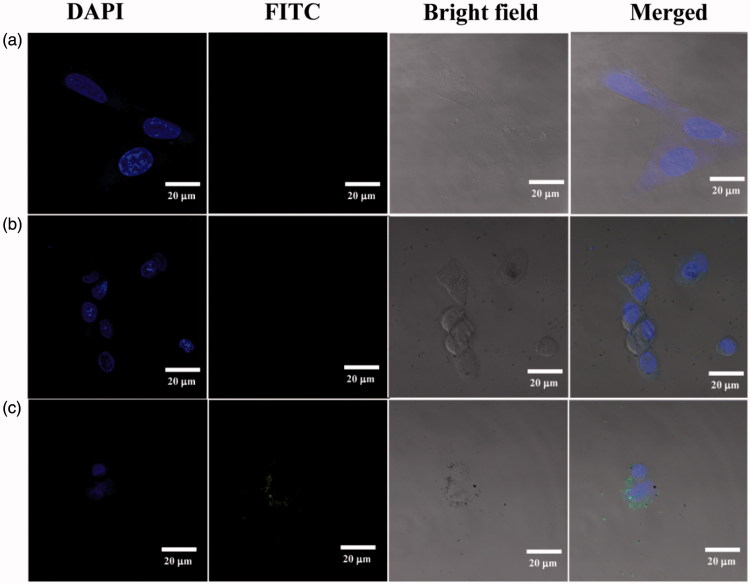
Confocal microscopy images of apoptotic C6 cells treated with PBS (a), Au DENPs (b), and duramycin-Au DENPs (c) at the Au concentration of 4 μM for 2 h, respectively.

Duramycin has been shown to have targeting specificity to PE on the surface of apoptotic cells. The conjugation of FI onto the dendrimers enabled us to analyze the cellular uptake of the duramycin-Au DENPs by flow cytometry and confocal microscopy. Through quantitative analysis of the ^1^H NMR spectra, the number of FI moiety modified onto each dendrimer was calculated to be 6.2 and 6.8 for the duramycin-Au DENPs and Au DENPs, respectively. From the flow cytometric analysis, it can be found that apoptotic C6 cells incubated with the duramycin-Au DENPs for 4 h display significantly higher fluorescence intensity than those treated with the Au DENPs (0.4 and 4.0 μM) (Figure S3(c)). In contrast, the normal C6 cells treated with the duramycin-Au DENPs exhibit similar fluorescence intensity to those treated with Au DENPs (Figure S3(d)). These data confirm that the duramycin-modified Au DENPs are able to specifically target apoptotic cancer cells presumably through specific PE interaction, in agreement with the literature (Elvas et al., 2016, 2017). Confocal microscopy was also used to further demonstrate the targeting specificity of the formed duramycin-Au DENPs (Figure 2). After 2 h incubation with the duramycin-Au DENPs (4.0 μM), apoptotic C6 cells display much stronger fluorescence signals than those treated with the control Au DENPs under similar conditions. On the basis of the confirmation of flow cytometric assay and confocal microscopic imaging, it can be concluded that the formed duramycin-Au DENPs are able to specifically target apoptotic cancer cells *via* receptor-mediated binding and endocytosis.

### Targeted SPECT/CT imaging of apoptotic C6 cells *in vitro*

**Figure 3.**
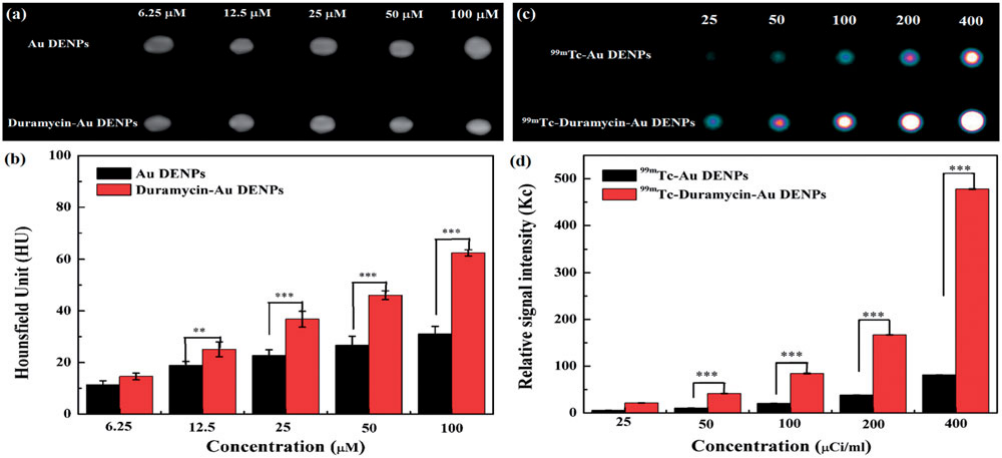
*In vitro* CT images (a) and the quantitative CT values (b) of apoptotic C6 cells treated with duramycin-Au DENPs or Au DENPs for 4 h at the different Au concentrations, respectively. *In vitro* SPECT images (c) and the quantitative SPECT signal intensity (d) of apoptotic C6 cells treated with ^99m^Tc-duramycin-Au DENPs or ^99m^Tc-Au DENPs for 4 h at the different radioactivity concentrations, respectively.

The feasibility to use the duramycin-Au DENPs for targeted apoptotic cancer cell CT imaging *in vitro* was next tested. Although the difference in the brightness of CT images between the cancer cells treated with different Au NPs is not obvious, quantitative CT value measurements show that apoptotic C6 cells treated with the duramycin-Au DENPs have a higher CT value than those treated with the Au DENPs at the given Au concentrations ([Au] = 20, 40, 60, 80, and 100 μM, respectively) (Figure 3(a, b)), while there is no obvious difference in the CT value of normal C6 cells treated with duramycin-Au DENPs and Au DENPs (Figure S6(a)). At the Au concentration of 100 μM, the CT contrast enhancement of apoptotic C6 cells using duramycin-Au DENPs is 1.8 and 2.0 times higher than that of normal C6 cells treated with the same NPs and that of apoptotic C6 cells treated with the Au DENPs, respectively. This suggests that the developed duramycin-Au DENPs enable targeted CT imaging of apoptotic C6 cells *in vitro*.

After ^99m^Tc radiolabeling, the applicability of ^99m^Tc-duramycin-Au DENPs for targeted SPECT imaging of apoptotic cancer cells *in vitro* was also validated. Similar to CT results above, the SPECT images of apoptotic C6 cells treated with the ^99m^Tc-duramycin-Au DENPs are much brighter than those of apoptotic C6 cells treated with the same NPs (Figure 3(c, d)), while normal C6 cells treated with the ^99m^Tc-duramycin-Au DENPs and ^99m^Tc-Au DENPs display similar SPECT signal intensity at the same radiodoses (Figure S6(b)). Quantitative SPECT signal intensity measurements at the ^99m^Tc dose of 400 μCi/mL shows that the SPECT signal enhancement of apoptotic C6 cells treated with the ^99m^Tc-duramycin-Au DENPs is 3.7 times higher than that of normal C6 cells treated with the same NPs and 5.8 times higher than that of apoptotic C6 cells treated with the ^99m^Tc-Au DENPs. This further demonstrates that the formed ^99m^Tc-duramycin-Au DENPs enable targeted SPECT imaging of apoptotic cancer cells *in vitro*.

### Targeted SPECT/CT imaging of an apoptotic tumor model *in vivo*

**Figure 4.**
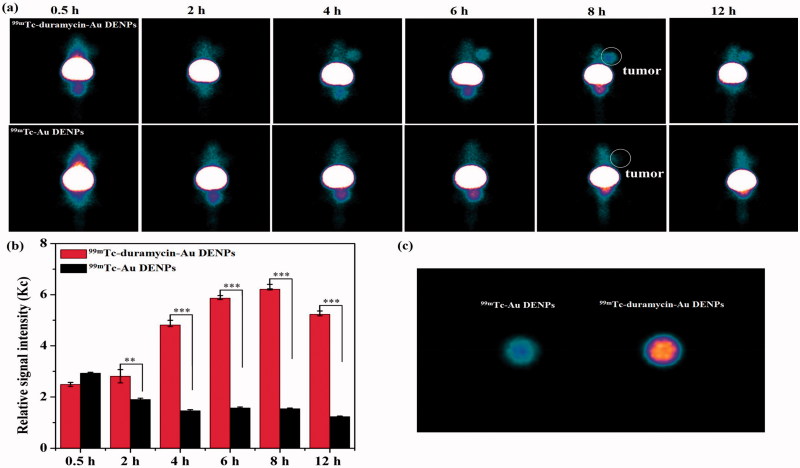
SPECT images (a) and tumor relative signal intensities (b) of the nude mice bearing C6 xenografted tumors after 3 days of DOX treatment at different time points post-intravenous injection of the ^99m^Tc-duramycin-Au DENPs or ^99m^Tc-Au DENPs. SPECT images of *ex vivo* tumors at 8 h post-injection (c). The white circle points to the tumor site.

With the success of *in vitro* SPECT and CT imaging of apoptotic cancer cells, we next evaluated the ^99m^Tc-duramycin-Au DENPs for SPECT/CT imaging of apoptotic cancer cells *in vivo* using a subcutaneous xenografted tumor model. As expected in the SPECT study, little accumulation of the ^99m^Tc-duramycin-Au DENPs or ^99m^Tc-Au DENPs was found in the tumors of mice before the DOX treatment during the investigated period (Figure S7), indicating that duramycin has no specificity to normal tumor cells. However, after 3 days of DOX treatment, a different *in vivo* behavior of ^99m^Tc-duramycin-Au DENPs in these mice can be observed. As shown in Figure 4, no visible tumor SPECT signal was observed for the mice injected with either the ^99m^Tc-duramycin-Au DENPs or ^99m^Tc-Au DENPs at 0.5 and 2 h post-injection. After that, the SPECT image of tumors treated with the ^99m^Tc-duramycin-Au DENPs is much brighter than that treated with the ^99m^Tc-Au DENPs at each time point, and the tumor SPECT signal intensity in the ^99m^Tc-duramycin-Au DENPs treated group reaches its peak value at 8 h post-injection and a clear tumor SPECT imaging can be still obtained at 12 h post-injection. In contrast, no tumor SPECT signal intensity changes were observed in the mice treated with the ^99m^Tc-Au DENPs during the same time period. To further confirm the significant difference in SPECT signal intensity of tumors treated with ^99m^Tc-duramycin-Au DENPs and ^99m^Tc-Au DENPs, the biodistribution of the multifunctional dendrimers and *ex vivo* tumors were scanned at 8 h post-injection (Figure S8). Distinctly, the majority of the multifunctional dendrimers are accumulated in the liver and spleen, while the heart, lung, tumor, kidney, intestines, stomach, and soft tissue have a relatively low accumulation of the nanoparticles. It should be noted that there is a relatively higher tumor uptake of the ^99m^Tc-duramycin-Au DENPs than ^99m^Tc-Au DENPs. This further confirms the specific targeting role mediated by the attached duramycin moiety onto the dendrimers.

**Figure 5.**
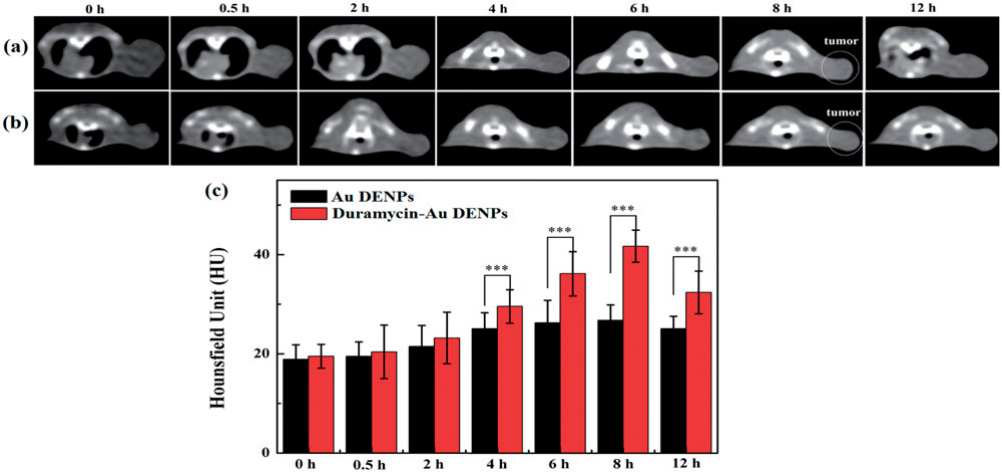
*In vivo* CT imaging images (a and b) and signal intensity (c) of tumors after intravenous injection of the duramycin-Au DENPs (a) or Au DENPs (b) at different time points post-injection. The white circle points to the tumor site.

Meanwhile, similar CT results were also obtained. As shown in Figure 5, the tumor CT value reaches the peak at 8 h for tumors treated with the duramycin-Au DENPs, while CT value in the tumors treated with the Au DENPs has no significant increase. Moreover, the tumor CT value treated with the duramycin-Au DENPs is 1.56 times higher than that treated with the Au DENPs (*p* < .001). Due to the duramycin-mediated specific targeting, the tumor CT value treated with the duramycin-Au DENPs is much higher than that treated with the nontargeted Au DENPs at the same time points (*p* < .01). The *in vivo* micro-CT imaging data suggest that the developed duramycin-Au DENPs can be applied as a nanoprobe for targeted CT imaging of early tumor response to treatment. Overall, our data indicate that the developed ^99m^Tc-duramycin-Au DENPs can be used as a nanoprobe for effective dual mode SPECT/CT imaging of tumor apoptosis after chemotherapy. Due to the fact that apoptosis exists universally in the treatment of various types of cancer cells, it is expected that the developed ^99m^Tc-duramycin-Au DENPs may also be used for targeted SPECT/CT imaging of many types of apoptotic cancer cells.

**Figure 6.**
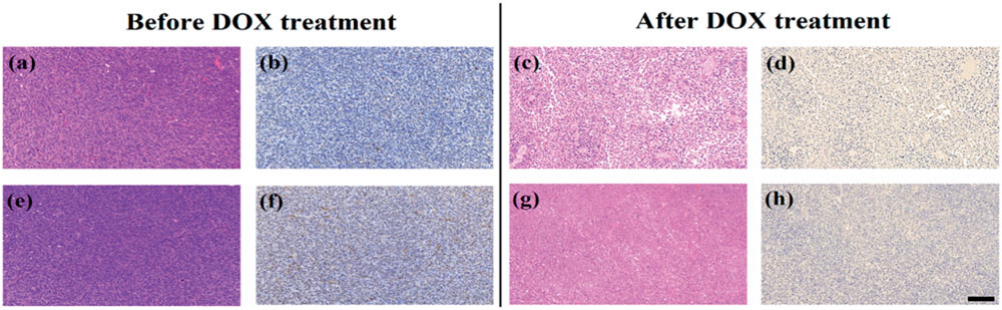
H&E staining (a, c, e, and g) and TUNEL assay (b, d, f, and h) of C6 xenografted tumors injected with ^99m^Tc-duramycin-Au DENPs (a, b, c, and d) or ^99m^Tc-Au DENPs (e, f, g, and h) before and after DOX treatment. The scale bar shown in all panels represents 200 μm.

H&E and TUNEL staining were further used to assess the apoptosis of C6 cell *in vivo* after DOX treatment (Figure 6). The results showed that tumor cells had no necrosis and apoptosis in the mice before DOX treatment. After 3 days of the treatment, necrotic regions and apoptosis tumor cells could be observed clearly both in the experimental and control groups, indicating that the apoptosis of C6 glioma cells were successfully induced by DOX. Finally, we investigated the potential toxicity of the multifunctional dendrimers after SPECT/CT imaging *via* H&E staining of the major organs including the heart, liver, spleen, lung, and kidney (Figure S9). No obvious organ damage or abnormalities can be seen. This suggests that the multifunctional dendrimers have good organ compatibility.

## Conclusion

We demonstrated a convenient approach to prepare multifunctional ^99m^Tc-labeled Au DENPs for targeted SPECT/CT imaging of tumor apoptosis induced after DOX treatment. G5 PAMAM dendrimers covalently modified with DOTA and PEGylated duramycin onto their surface could be used to entrap Au NPs and labeled with ^99m^Tc *via* DOTA chelation. The developed multifunctional {(Au^0^)_200_-G5.NHAc-^99m^Tc-DOTA-FI-*m*PEG-(PEG-duramycin)} DENPs display excellent stability and cytocompatibility in the investigated concentration range. They could be used as a nanoprobe for targeted dual mode SPECT/CT imaging of apoptotic cancer cells *in vitro* and a tumor model *in vivo*. The developed dendrimer-based nanoprobe could be used for early detection of apoptosis after chemotherapy.

## Supplementary Material

Supplemental Material
