# Supplementary material for: SPECT/CT imaging of chemotherapy-induced tumor apoptosis using 99mTc-labeled dendrimer-entrapped gold nanoparticles
Source: Drug Deliv. 2018 Jun 5;25(1):1384–93. doi: 10.1080/10717544.2018.1474968 (PMC6058576; doi:10.1080/10717544.2018.1474968)
Supplement: Supplemental Material [file IDRD_A_1474968_SM0129.docx]

**Supporting Information**

**SPECT/CT imaging of chemotherapy-induced tumor apoptosis using ^99m^Tc-labeled dendrimer-entrapped gold nanoparticles**

Yan Xing^1, 2§^, Jingyi Zhu^3, 4§^, Lingzhou Zhao^2§^, Zhijuan Xiong^3^, Yujie Li^2^, San Wu^2^, Gitasha Chand^2^, Xiangyang Shi^3,^ * and Jinhua Zhao^1, 2^*

^1^ Department of Nuclear Medicine, Shanghai General Hospital of Nanjing Medical University, Shanghai 200080, People’s Republic of China

^2^ Department of Nuclear Medicine, Shanghai General Hospital, Shanghai Jiao Tong University School of Medicine, Shanghai 200080, People’s Republic of China

^3^ State Key Laboratory for Modification of Chemical Fibers and Polymer Materials, College of Chemistry, Chemical Engineering and Biotechnology, Donghua University, Shanghai 201620, People’s Republic of China

^4^ School of Pharmaceutical Science, Nanjing Tech University, Nanjing, 211816, People’s Republic of China

________________________________________________________

* Corresponding authors: zhaojinhua1963@126.com (J. Zhao) and xshi@dhu.edu.cn (X. Shi)

^§^These authors equally contributed to this work.

**Part of experimental section**

**Characterization techniques**

UV-Vis spectra were measured using a Lambda 25 UV-Vis spectrometer (Perkin Elmer, Waltham, USA). ^1^H NMR spectra were recorded by a Bruker DRX 400 nuclear magnetic resonance spectrometer. All samples were dissolved in D2O before experiments. Leeman Prodigy inductively coupled plasma-optical emission spectroscopy (ICP-OES, Hudson, NH) was used to determine the Au composition of Au PENPs. The size and morphology of Au DENPs were obtained using a JEOL 2010F analytical electron microscope (JEOL, Tokyo, Japan). Malvern Zetasizer Nano ZS model ZEN3600 (Worcestershire, UK) was used to perform the dynamic light scattering (DLS) and zeta potential measurements using a standard 633 nm laser. Au DENPs and Omnipaque solution ((iohexol 300, GE Healthcare)) with different Au or I concentrations were scanned *via* a GE LightSpeed VCT imaging system (GE Medical Systems) for the measurement of X-ray attenuation property. CT images were gained by a GE LightSpeed VCT imaging system (GE Medical Systems, Milwaukee, WI). Contrast enhancement in Hounsfield units (HU) for each sample was measured.

***In vitro* stability assay**

The colloidal stability of the duramycin-Au DENPs was evaluated by UV-vis spectroscopy under varying pH and temperature conditions according to our previous protocol described in the literature (Li et al., 2016). Moreover, the formed ^99m^Tc-duramycin-Au DENPs (200 μL, 74 MBq/ml) were incubated in 2 mL of 0.9% saline at room temperature for 8 h to evaluate the stability of radiolabeled nanoparticles *in vitro*. The results were analyzed by a thin-layer chromatogram scanner (Bioscan Inc., Tucson, AZ) with silica gel-coated fiber glass sheets (Macherey-Nagel, GmbH & Co. KG, Düren, Germany) to measure the radiochemical purities at different time points (5 min, 2, 4, 6, and 8 h, respectively). The stability of ^99m^Tc-Au DENPs without duramycin modification was also evaluated using the similar procedure.

**Cells and animals**

C6 cells were cultured in DMEM/F12 with 4% FBS and RPMI-1640 with 10% FBS, respectively. All culture medium were supplemented with 100 U/ml streptomycin and 100 U/ml penicillin. Cells were cultured in a humidified incubator (5% CO_2_ and 37°C) and the culture medium was refreshed at 1-2 day intervals.

Six-week-old BALB/c female nude mice (21-23 g) were provided by Shanghai Slac Laboratory Animal Center and raised in the Animal Laboratory of Shanghai General Hospital. All animal experiments were performed according to protocols established by the ethics committee of Shanghai General Hospital. The mice were inoculated subcutaneously with 1 × 10^6^ C6 cells/mouse in the right side of flank. When the tumors reached a volume of 0.5-0.8 cm^3^ at approximately 3 weeks postinjection, these mice would be ready for animal experiment use.

**Cytotoxicity assay**

CCK-8 assays and morphology observation of C6 cells were performed to assess the cytocompatibility of the duramycin-Au DENPs or Au DENPs at different Au concentrations (0, 10, 50, 100 and 200 μM) according to protocols described in the literature (Luo et al., 2016).

**Part of results and discussion**

**Stability of the {(Au^0^)_200_-G5.NHAc-^99m^Tc-DOTA-*m*PEG-(PEG-duramycin)} DENPs**

The good stability of prepared Au NPs is essential for their further biomedical applications. Generally, the absorption features of Au NPs are directly relevant to their stabilities, hence, we used UV-vis spectrometry to evaluate the colloidal stability of duramycin-Au DENPs under different temperature and pH conditions (Figure S2c and d). The results show that no apparent absorption feature changes of the duramycin-Au DENPs can be found after being stored in water for a period of 7 days, suggesting that the formed Au NPs have good stability under the given pH and temperature conditions.

Meanwhile, prior to SPECT imaging application, the radiochemical stability of ^99m^Tc-labeled Au DENPs should be investigated by measuring their radiochemical purities at different time points (Figure S3a). The data reveal that both ^99m^Tc-duramycin-Au DENPs and ^99m^Tc-Au DENPs have satisfactory stabilities within 8 h *in vitro* after exposure to PBS solution, and at least 90% of the ^99m^Tc-labeled dendrimers still keep the original structure without ^99m^Tc separation from the dendrimer backbones. This suggests that the ^99m^Tc labeling onto the dendrimer is pretty stable, ensuring the further SPECT imaging application *in vivo*.

**Cytotoxicity assay**

The cytotoxicity of the Au DENPs prior to ^99m^Tc labeling was assessed by CCK-8 assays (Figure S3b). Clearly, the viability of C6 cells treated with duramycin-Au DENPs or Au DENPs at the concentration up to 200 μM still remains at 90% or above and does not have significant difference when compared with that of C6 cells treated with PBS (p > 0.05). The cytocompatibility was further examined by observation of the morphology of cells treated with duramycin-Au PENPs (Figure S4). It is shown that no apparent morphological changes occur even at the Au concentration up to 200 μM in comparison to the control cells treated with PBS, which suggests that the formed Au NPs display good cytocompatibility in the studied concentrations.

**X-ray attenuation property**

For CT imaging applications, we compared the X-ray attenuation property of the developed duramycin-Au DENPs and clinically used small molecules Omnipaque (Figure S5). Evidently, the brightness of CT images increases with Au or I concentration, while the duramycin-Au DENPs have significantly larger CT values than Omnipaque under the same Au or I concentrations. In both cases, the X-ray attenuation intensity increases with the Au or I concentration, and the increasing trend of the duramycin-Au DENPs that is considerably higher than that of Omnipaque, suggesting that the formed Au DENPs possess a better X-ray attenuation property than Omnipaque.

**Reference**

Li X, Xiong Z, Xu X, et al. (2016). ^99m^Tc-Labeled Multifunctional Low-Generation Dendrimer-Entrapped Gold Nanoparticles for Targeted SPECT/CT Dual-Mode Imaging of Tumors. ACS Appl Mater Interfaces 8**:** 19883-91.

Luo Y, Zhao L, Li X, et al. (2016). The design of a multifunctional dendrimer-based nanoplatform for targeted dual mode SPECT/MR imaging of tumors. J Mater Chem B 4**:** 7220-5.

**Table S1**. Zeta potentials and hydrodynamic size of the duramycin-Au DENPs and Au DENPs.

| Samples | Hydrodynamic  size (nm) | Polydispersity  index | Zeta potential  (mV) |
| --- | --- | --- | --- |
| duramycin-Au DENPs | 327.00 | 0.36 | 11.93 |
| Au DENPs | 287.90 | 0.54 | -0.11 |


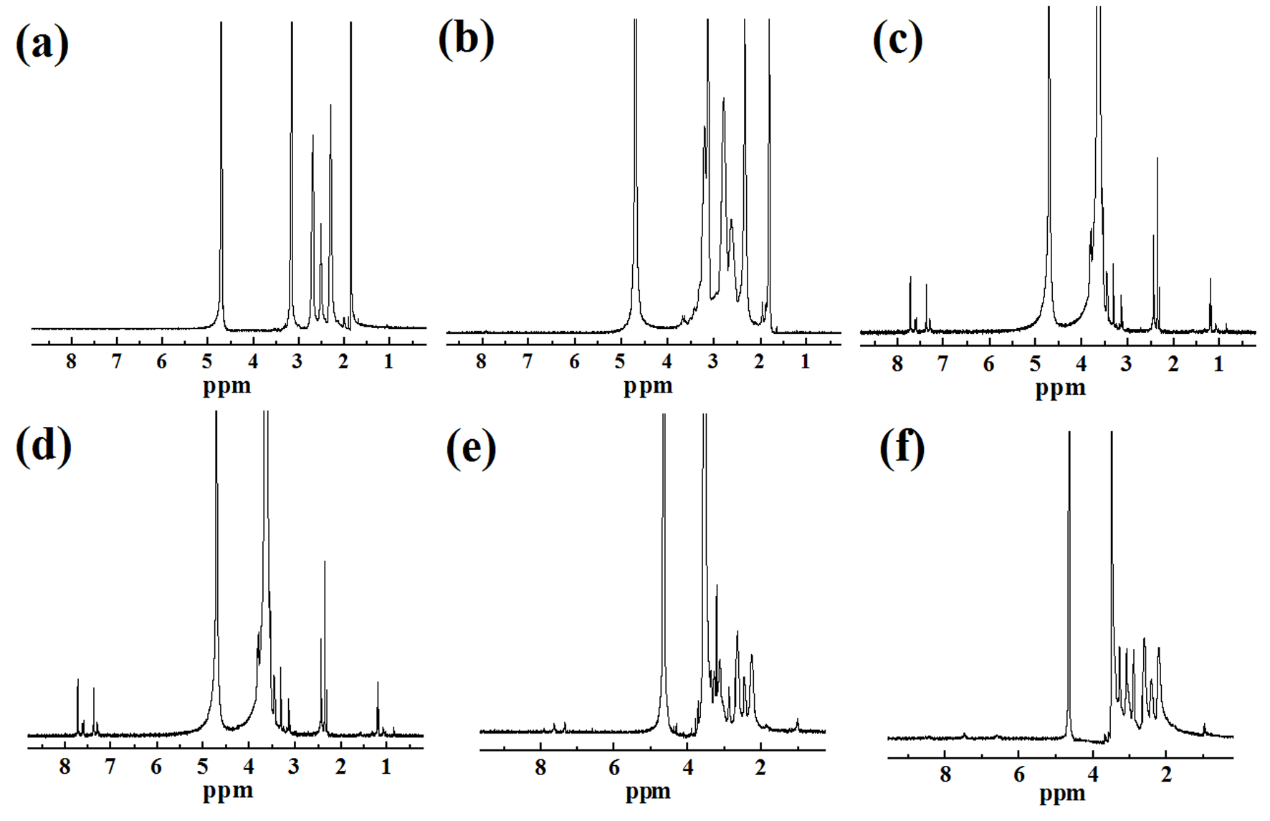


**Figure S1. ^1^**H NMR spectra of G5.NHAc (a), G5.NHAc-DOTA (b), duramycin-PEG-COOH (c), G5.NH_2_-DOTA-(PEG-duramycin) (d), G5.NH_2_-DOTA-FI-*m*PEG-(PEG-duramycin) (e) and G5-NH_2_-DOTA-FI-*m*PEG (f), respectively.


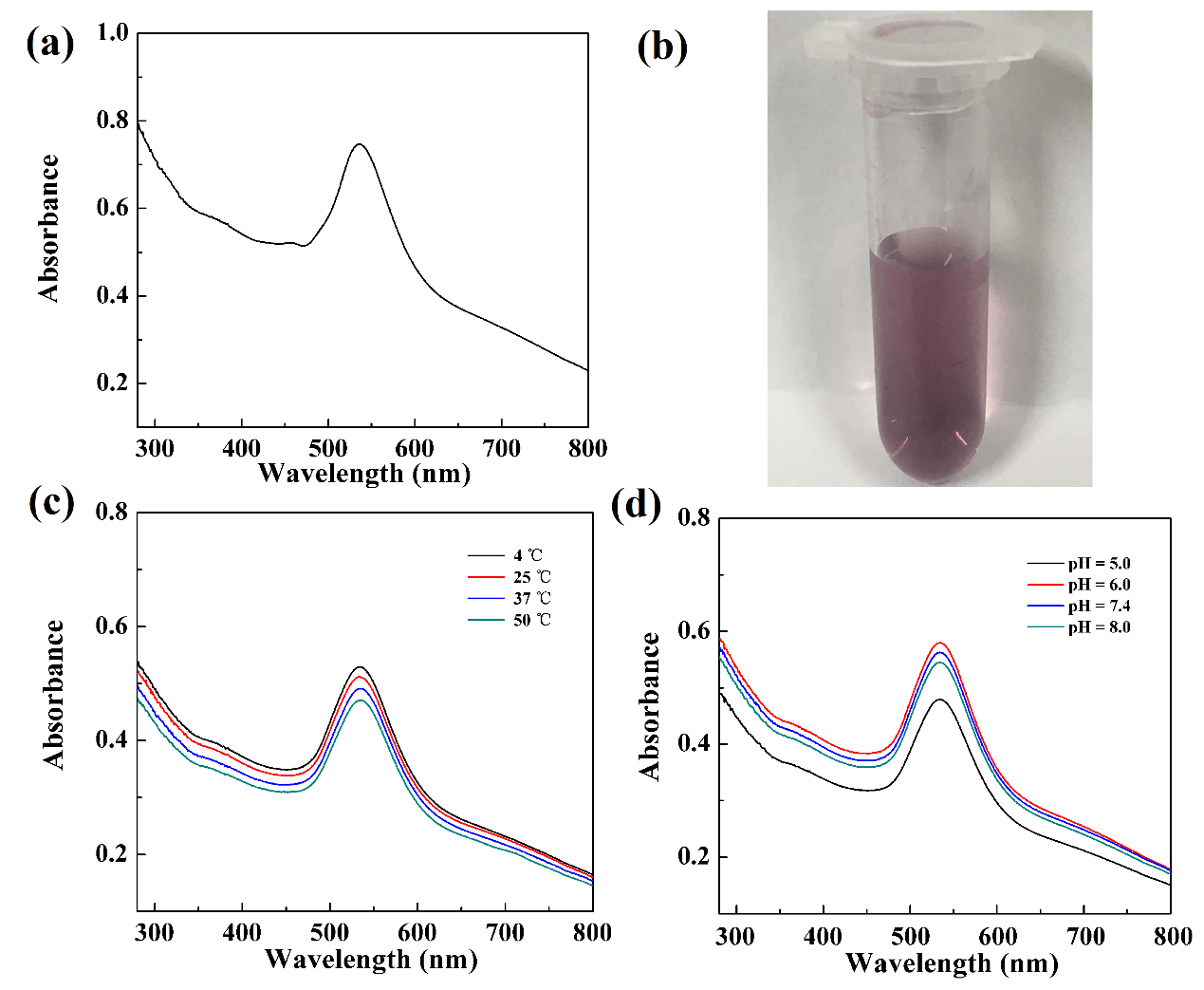


**Figure S2**. UV-vis spectra of the duramycin-Au DENPs (a) dispersed in water at different temperature (c) and pH (d) conditions; (b) shows the photograph.


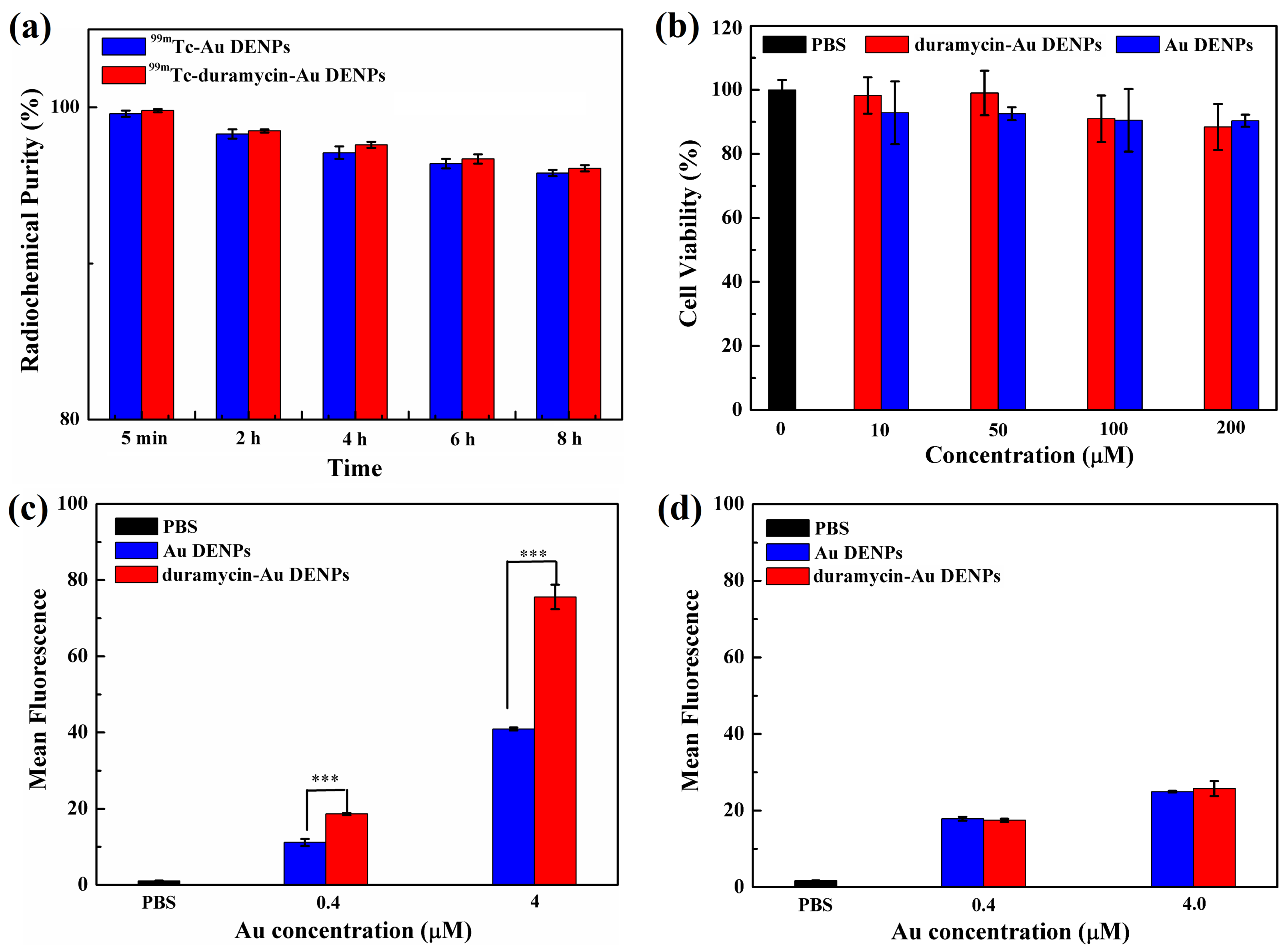


**Figure S3**. (a) Radiochemical purity of the ^99m^Tc-duramycin-Au DENPs and ^99m^Tc-Au DENPs exposed to PBS at room temperature for different time periods. (b) CCK-8 assay of normal C6 cells treated with the duramycin-Au DENPs or Au DENPs at different Au concentrations for 24 h, respectively. Flow cytometric analysis of the apoptotic (c) and normal C6 cells (d) incubated with duramycin-Au DENPs or Au DENPs at different Au concentrations for 4 h, respectively. The cells treated with PBS were used as controls, and the data were expressed as mean ± SD (n = 3).


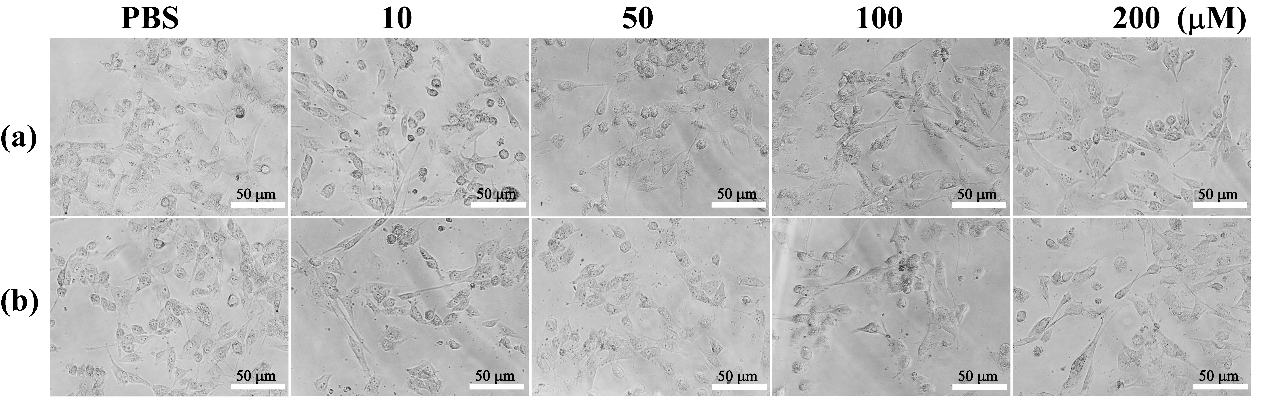


**Figure S4**. Micrographs of normal C6 cells treated with the duramycin-Au DENPs (a) and Au DENPs (b) at the Au concentrations of 0, 10, 50, 100 and 200 μM for 24 h, respectively. The scale bar in each panel represents 50 μm.


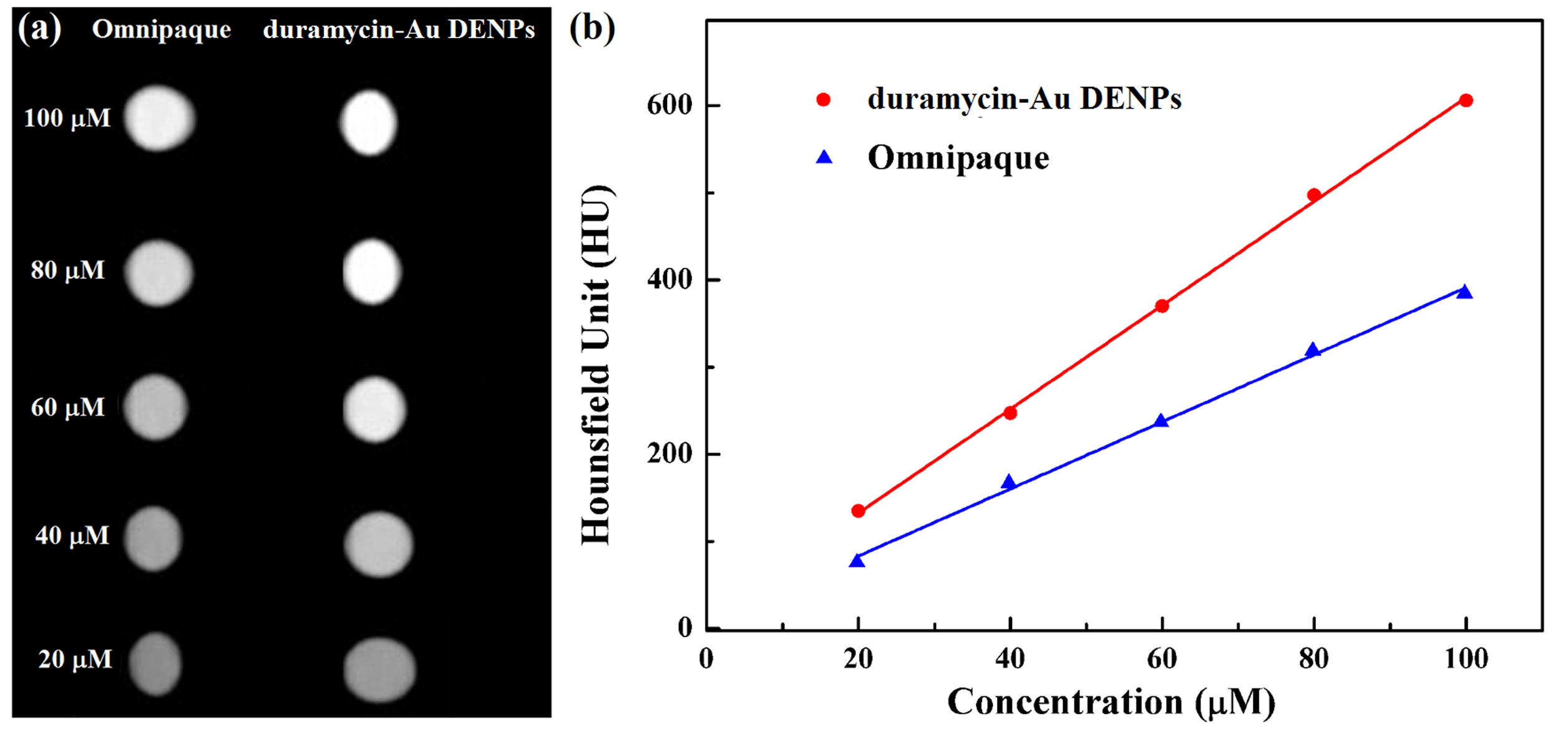


**Figure S5**. (a) CT images and (b) X-ray attenuation intensity (HU) of duramycin-Au DENPs and Omnipaque at different concentrations of Au or iodine.


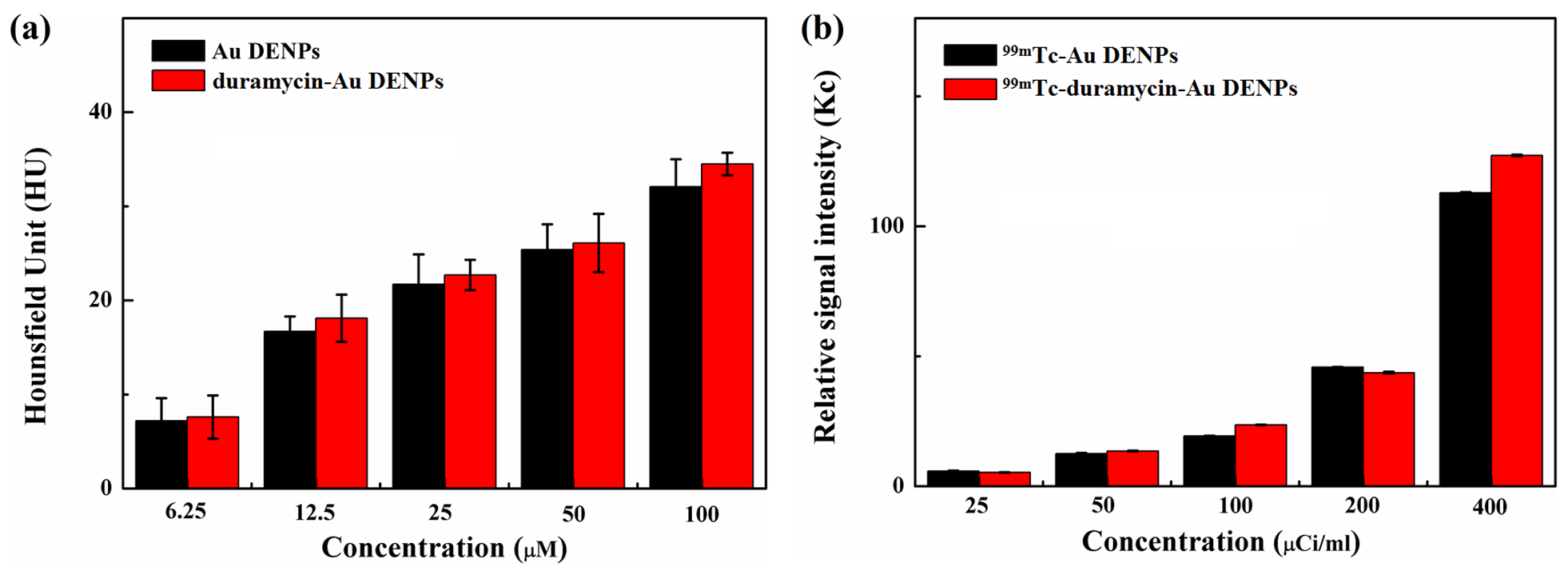


**Figure S6**. The quantitative CT values (a) of normal C6 cells treated with the duramycin-Au DENPs or Au DENPs for 4 h at the different Au concentrations, respectively. The quantitative SPECT signal intensity (b) of normal C6 cells treated with the ^99m^Tc-duramycin-Au DENPs or ^99m^Tc-Au DENPs for 4 h at the different radioactive concentration, respectively.


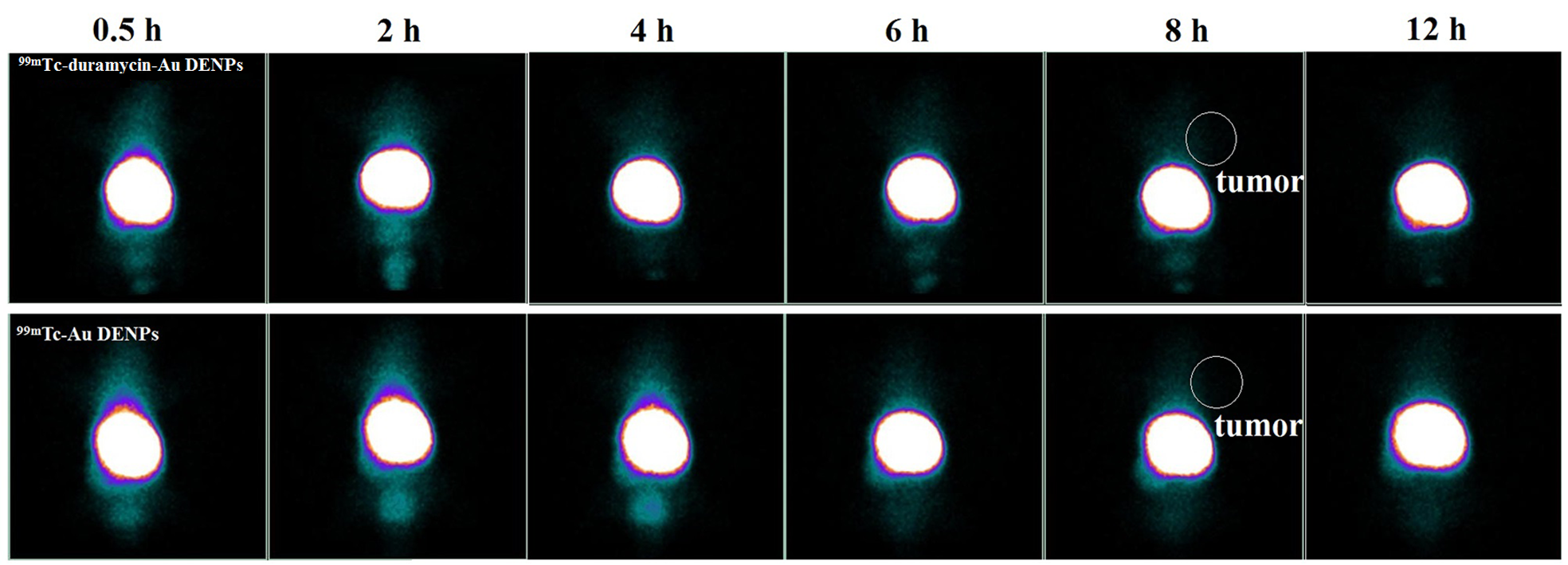


**Figure S7**. SPECT images of the nude mice bearing C6 xenografted tumors before DOX treatment at different time points post intravenous injection of the ^99m^Tc-duramycin-Au DENPs or ^99m^Tc-Au DENPs. The white circle points to the tumor site.


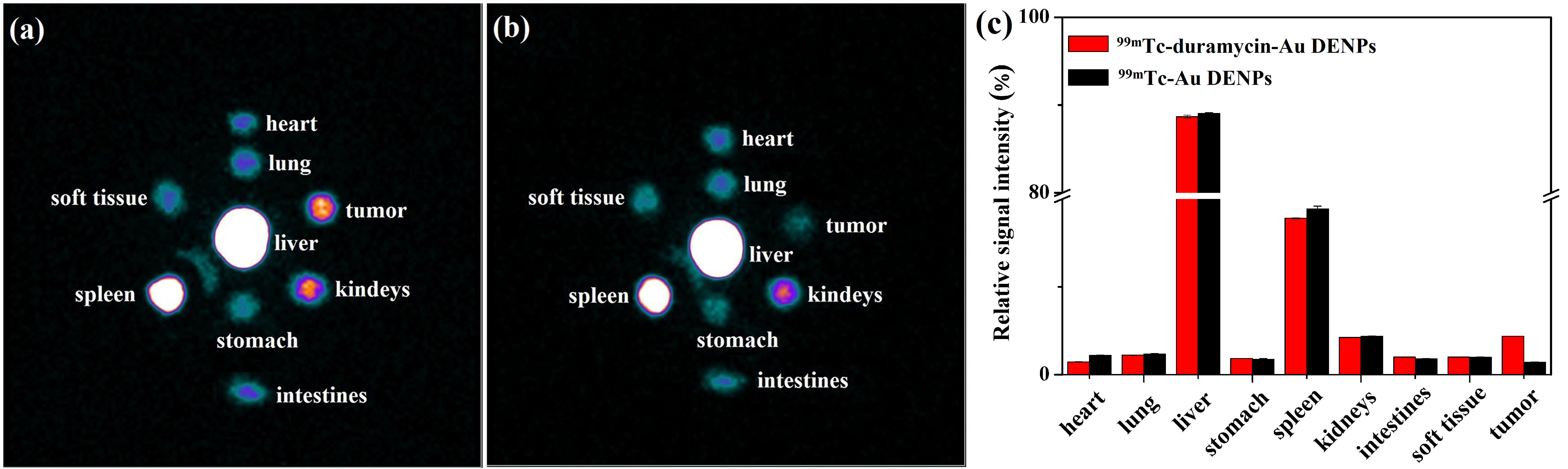


**Figure S8.** The biodistribution of ^99m^Tc-duramycin-Au DENPs (a) and ^99m^Tc-Au DENPs (b), and their relative signal intensities of different organs (c) at 8 h postinjection.

**
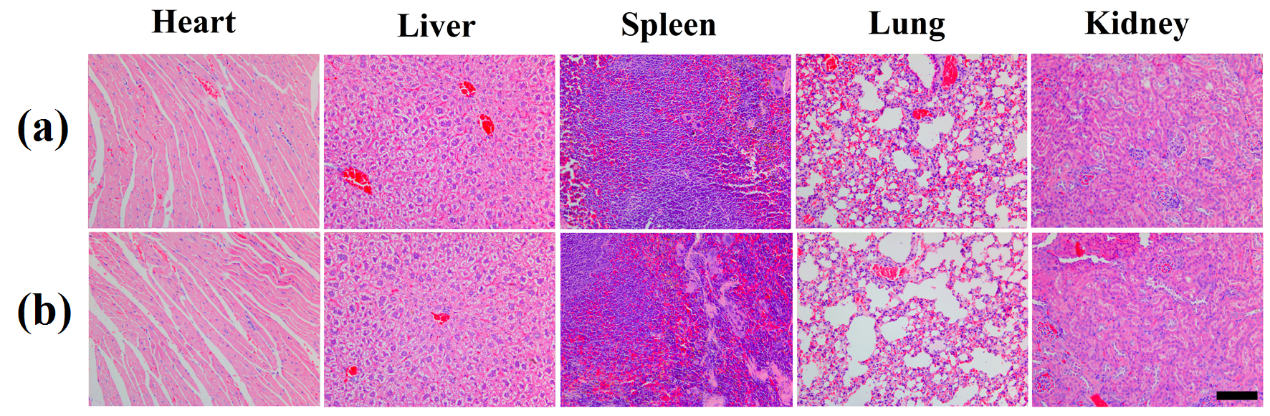
**

**Figure S9.** H&E stained tissue sections of major organs including heart, liver, spleen, lung, and kidney from survived mice injected with ^99m^Tc-duramycin-Au DENPs (a) or ^99m^Tc-Au DENPs (b), respectively. The scale bar (applied for all panels) represents 200 μm.
